# Repair of alveolar cleft bone defects by bone collagen particles combined with human umbilical cord mesenchymal stem cells in rabbit

**DOI:** 10.1186/s12938-020-00800-4

**Published:** 2020-08-03

**Authors:** Xue-Cheng Sun, Hu Wang, Jian-hui Li, Dan Zhang, Li-Qiang Yin, Yu-Fang Yan, Xu Ma, Hong-Fei Xia

**Affiliations:** 1 Reproductive and Genetic Center of National Research Institute for Family Planning, Beijing, 10081 China; 2 Graduate Schools, Peking Union Medical College, Beijing, 100730 China; 3 Yantai Zhenghai Bio-Tech Co., Ltd, Shandong, 264006 China

**Keywords:** Alveolar cleft, Bone collagen particles, HUC-MSCs (human umbilical cord mesenchymal stem cells), Micro-focus computerised tomography (micro-CT)

## Abstract

**Background:**

Alveolar cleft is a type of cleft lip and palate that seriously affects the physical and mental health of patients. In this study, a model of the alveolar cleft phenotype was established in rabbits to evaluate the effect of bone collagen particles combined with human umbilical cord mesenchymal stem cells (HUC-MSCs) on the repair of alveolar cleft bone defects.

**Methods:**

A model of alveolar clefts in rabbits was established by removing the incisors on the left side of the upper jaw bone collagen particles combined with HUC-MSCs that were then implanted in the defect area. Blood biochemical analysis was performed 3 months after surgery. Skull tissues were harvested for gross observation, and micro-focus computerised tomography (micro-CT) analysis. Tissues were harvested for histological and immunohistochemical staining. The experiments were repeated 6 months after surgery.

**Results:**

Bone collagen particles and HUC-MSCs showed good biocompatibility. Bone collagen particles combined with HUC-MSCs were markedly better at inducing bone repair and regeneration than bone collagen particles alone.

**Conclusions:**

Combining HUC-MSCs with bone collagen particles provides a simple, rapid and suitable method to fill a bone defect site and treat of alveolar cleft bone defects.

## Background

Alveolar cleft is a common disease that not only affects the normal eruption of teeth and the development of the jaws, but also affects the physical and mental health of patients [1]. Therefore, it is very important to establish a repeatable animal model of alveolar cleft that is similar to human alveolar clefts disease. This method can provide a theoretical basis for the occurrence and development of the disease, as well as provide a good scientific research foundation for the repair of alveolar clefts.

The most commonly used model animals are primates [2, 3], sheep [4], canines [5], felids [6], rodents and rabbits [7, 8]. Rabbits have the advantage of a short growth cycle, simple diet and a low cost. Moreover, compared with other model animals, rabbits are of moderate size, gentle temperament and easy to handle. Studies in our laboratory have also proved that rabbits can be used to establish animal models of the alveolar cleft [9]. Therefore, rabbits were selected as experimental animals to establish an animal model of alveolar clefts in the present study.

Alveolar clefts are often associated with cleft lip. Although cleft lip can be corrected by surgical suture, there is yet no effective treatment for alveolar clefts caused by bone defects. At present, the treatment methods for alveolar clefts can be divided into distraction osteogenesis and bone grafting.

Distraction osteogenesis refers to a technology of bone correction or repair in which physical traction force is applied in a specific direction to partially or completely detach biological tissue. Thereafter, this gap is gradually filled with new bone. Binqer et al. used distraction osteogenesis to correct alveolar clefts [10]. Although this method avoids the immunogenicity of the foreign implanted tissue, the procedure is complicated, requires a long treatment period, and requires two surgeries to place and remove the retractor. Therefore, distraction osteogenesis is not an optimal treatment.

Bone materials commonly used in bone grafting include autogenous bone, allogeneic bone, and tissue-engineered bone. Boyne et al. repaired oronasal fistulas by inserting a small amount of cancellous bone from the autogenous ilium into the fractures [11]. This method is considered the gold standard for the clinical repair of alveolar clefts [12, 13]. Nevertheless, autologous bone grafts are associated with donor trauma and deformity, which is not an ideal repair method. Nique et al. used allograft bones to treat alveolar clefts, and postoperative imaging showed that the tooth successfully erupted and grew into the graft bone; however, this process required at least 3 months [14]. El Deeb et al. used hydroxyapatite in the gaps of the alveolar clefts and found no tooth eruption [15]. Although allogeneic bone allograft or artificial bone, avoid donor-site deformity, there is a risk of immune rejection and disease transmission. With the development of tissue engineering technology, the application of tissue engineering bones to repair alveolar fractures is no longer a problem. Currently, the most widely used scaffold materials are collagen [16], hydroxyapatite [17], calcium sulphate [18], calcium phosphate cement [19], and bioactive glass [20]. However, a single material alone cannot obviously achieve the effect of repair alveolar clefts due the complex structure and function of bone tissue. As such, combining scaffold materials with bone growth factors or stem cells that induce bone regeneration is commonly used to repair alveolar clefts. Compared with other bone growth factors, stem cells have the advantages of easy access and fast reproduction [22]. Commonly used stem cells are bone marrow mesenchymal stem cells [21], umbilical cord mesenchymal stem cells [22, 23], and embryonic stem cells. Compared with stem cells derived from bone marrow, stem cells derived from the umbilical cord have the advantages of low immunogenicity, rapid proliferation, wide availability and low ethical concerns [23, 24]. Studies have shown that HUC-MSCs combined with collagen scaffolds can be used to treat chronic spinal cord injury in dogs [25]. In this study, bone collagen granules prepared by the decellularisation and degreasing of bovine cancellous bone were used as scaffold materials. The main ingredients are collagen and hydroxyapatite, which preserve the bone’s natural three-dimensional porous structure and reduce its immunogenicity. On this basis, composite human umbilical cord-derived mesenchymal stem cells were used to repair alveolar fissures in the present study.

The purpose of this study is to investigate the feasibility and effectiveness of bone collagen particles inoculated with HUC-MSCs in a rabbit model of alveolar clefts. The results indicate that HUC-MSCs combined with bone collagen particles may be a reliable alternative therapy for the repair of alveolar bone defects.

## Results

### Blood analysis

**Fig. 1:**
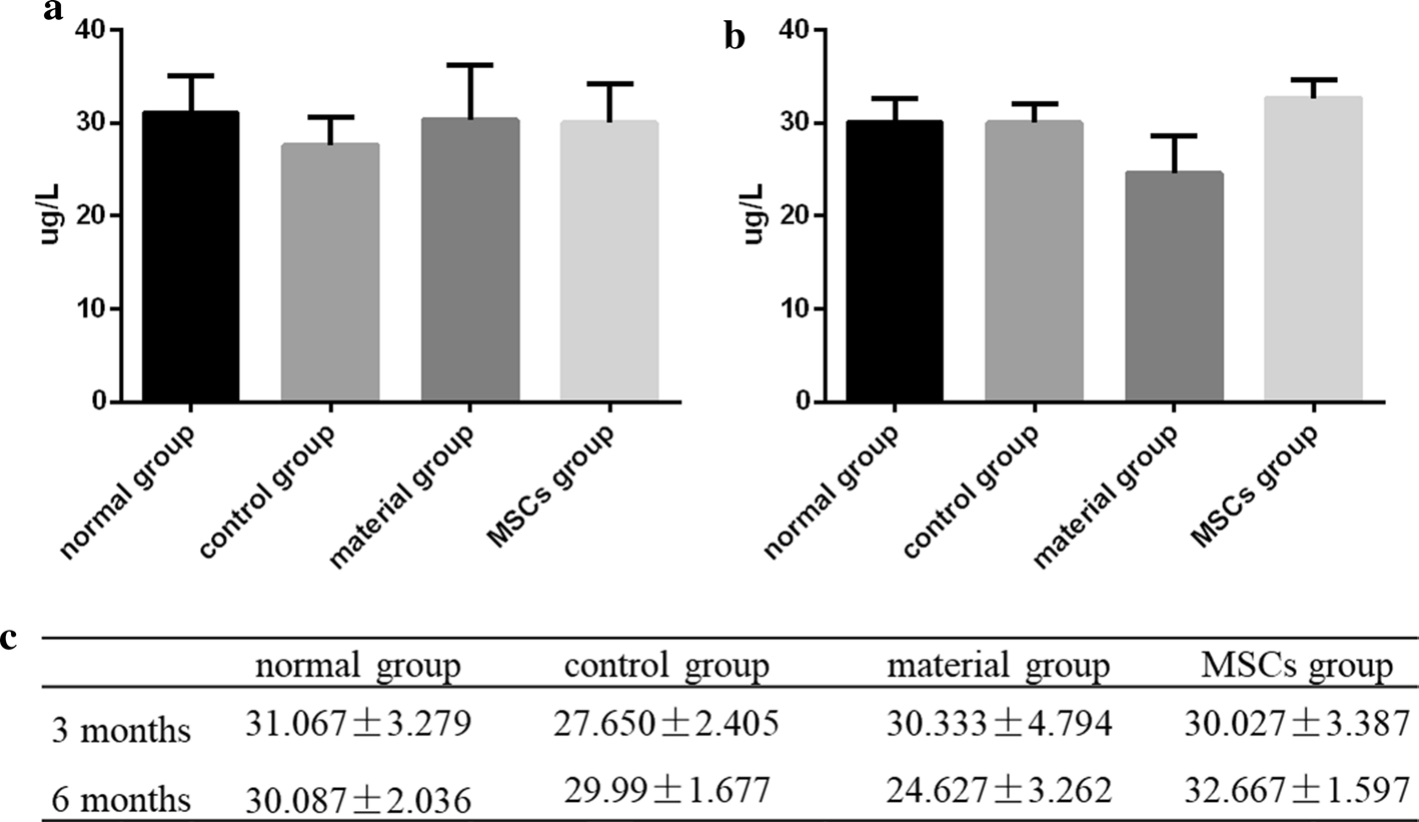
**a** The content of serum BGP in each group at 3 months. **b** The content of serum BGP in each group at 6 months. **c** The values of BGP results at 3 and 6 months postoperatively (mean ± SD)

Routine blood (Table 1), liver function (Table 2), renal function (Table 3) and bone Gla protein (BGP) (Fig. 1a) in each group (normal, control, material and MSCs groups) were measured 3 months after surgery. Routine blood (Table 4), liver function (Table 5), renal function (Table 6) and bone Gla protein (BGP) (Fig. 1b) in each group (normal, control, material and MSCs groups) were measured again 6 months after surgery. The values of control, material and MSCs groups were compared with those of the normal group. The blood routine results showed that the neutrophil granulocyte (NEUT) content increased whilst the lymphocyte (LYM) content decreased, in the control group 3 months after surgery, and were all close to normal at 6 months. The levels of C-reactive protein (CRP), eosinophil (EO) and NEUT in the material group increased at 3 months, whilst the content of the LYM decreased, and all were close to normal at 6 months. The CRP levels in the MSCs group decreased after 6 months, and other indicators showed no significant abnormality. Liver function results showed that the levels of alanine aminotransferase (ALT) and aspartate aminotransferase (AST) in the control group were higher than those in the normal group at 3 and 6 months. The ALT and AST levels in the material group were higher than those in the normal group at 3 months and normal at 6 months. No significant abnormalities were found in the MSCs group. The renal function results showed that CR levels in the control group, the material group and the MSCs group increased significantly at 3 months and were close to normal group at 6 months. The blood urea nitrogen (BUN) levels in the control and MSCs groups increased significantly at 3 months and were close to the normal group at 6 months.

**Table 1:** Blood routine test results at 3 months postoperatively

Detection index	Unit	Normal group	Control group	Material group	MSCs group
RBC	10^12^/L	5.727 ± 0.460	5.757 ± 0.802	5.660 ± 0.067	4.803 ± 0.759
HCT	%	36.467 ± 2.120	37.867 ± 2.839	37.300 ± 1.631	31.167 ± 4.619
RDW-CV	10^9^/L	13.033 ± 0.873	14.400 ± 0.589	13.333 ± 0.262	15.267 ± 2.864
RDW-SD	%	29.400 ± 2.192	33.767 ± 3.420	30.867 ± 1.793	35.367 ± 7.014
MCV	fL	63.800 ± 1.344	66.367 ± 4.739	65.133 ± 2.593	64.900 ± 1.283
HBG	g/L	117.667 ± 6.182	123.000 ± 13.491	121.000 ± 2.944	103.333 ± 16.680
MCH	pg	20.600 ± 0.980	21.433 ± 0.910	21.100 ± 0.455	21.433 ± 0.450
MCHC	g/L	323.000 ± 10.033	323.667 ± 10.965	324.667 ± 8.179	330.667 ± 13.123
WBC	10^9^/L	13.170 ± 2.788	8.680 ± 2.961	10.037 ± 1.848	8.603 ± 1.062
LYM#	10^9^/L	4.685 ± 0.672^**##**^	2.243 ± 0.452**	2.318 ± 0.183**	3.195 ± 0.428*
LYM%	%	36.080 ± 2.401	28.633 ± 9.420	23.670 ± 3.746	37.130 ± 2.007
NEUT#	10^9^/L	7.547 ± 2.054	5.652 ± 2.275	6.606 ± 1.369	4.589 ± 0.761
NEUT%	%	56.617 ± 3.356	63.050 ± 6.846	65.517 ± 2.569	53.220 ± 4.038
MONO#	10^9^/L	0.664 ± 0.132	0.579 ± 0.329	0.780 ± 0.267	0.492 ± 0.141
MONO%	%	5.120 ± 0.889	5.923 ± 2.294	7.573 ± 1.156	5.740 ± 1.511
EO#	10^9^/L	0.229 ± 0.022	0.153 ± 0.070	0.320 ± 0.113	0.235 ± 0.047
EO%	%	1.800 ± 0.318	1.710 ± 0.513	3.090 ± 0.565	2.760 ± 0.556
BASO	10^9^/L	0.045 ± 0.017	0.052 ± 0.033	0.016 ± 0.003	0.092 ± 0.043
BASO%	%	0.383 ± 0.210	0.683 ± 0.363	0.150 ± 0.037	1.150 ± 0.689
PLT	10^9^/L	153.333 ± 35.188	171.667 ± 22.867	141.000 ± 24.536	127.667 ± 8.498
PDW	%	15.700 ± 0.294	15.967 ± 0.403	15.967 ± 0.478	15.533 ± 0.047
MPV	fL	7.167 ± 0.411	7.133 ± 0.249	7.300 ± 0.653	6.767 ± 0.450
PLCR	%	14.267 ± 4.606	14.833 ± 3.167	14.300 ± 3.511	10.567 ± 1.761
PCT	%	0.041 ± 0.021	0.028 ± 0.011	0.038 ± 0.030	0.013 ± 0.005
CRP	mg/l	6.400 ± 1.425	12.433 ± 4.488	6.870 ± 3.556	3.143 ± 2.191^**#**^

Mean SD values were calculated for each group

*RBC* red blood cell, *HCT* haematocrit, *RDW-CV* red blood cell volume distribution width, *RDW-SD* red blood cell distribution width, *MCV* mean corpuscular volume, *HBG* haemoglobin, *MCH* mean corpuscular haemoglobin, *MCHC* mean corpuscular haemoglobin concentration, *WBC* white blood cell, *LYM* lymphocyte, *NEUT* neutrophile granulocyte, *MONO* monocyte, *EO* eosinophil, *BASO* basophil, *PLT* platelet, *PDW* platelet distribution width, *MPV* mean platelet volume, *PLCR* platelet-large cell ratio, PCT platelet volume, *CRP* C reactive protein

^*^The statistical difference between each group and the normal group. ^#^ The statistical difference between each group and the control group. *, ^#^ P<0.05; **, ^##^ P<0.01

**Table 2:** Liver function test results in blood at 3 months postoperatively

Detection index	Unit	Normal group	Control group	Material group	MSCs group
ALT	IU/L	44.533 ± 5.424	82.167 ± 23.572	62.333 ± 7.903	41.400 ± 11.051^#^
AST	IU/L	30.567 ± 6.215	76.000 ± 48.880	44.833 ± 29.613	16.167 ± 2.595
ALP	IU/L	47.000 ± 17.705	59.767 ± 10.700	67.400 ± 13.983	23.867 ± 5.188
TP	g/l	52.533 ± 0.694	58.000 ± 3.827	59.133 ± 2.845	48.900 ± 7.920
ALB	g/l	33.500 ± 0.424	38.633 ± 3.307	36.667 ± 1.066	27.900 ± 7.896
GLB	g/l	19.000 ± 1.042	19.400 ± 2.825	22.467 ± 3.561	21.000 ± 2.140
A/G		1.767 ± 0.119	2.040 ± 0.381	1.680 ± 0.299	1.347 ± 0.411
TBIL	Umol/L	9.247 ± 1.036	8.080 ± 1.408	8.917 ± 2.806	6.953 ± 0.345
DBIL	Umol/L	5.093 ± 0.718	3.870 ± 0.388	4.817 ± 0.749	2.977 ± 0.310*
IBIL	Umol/L	4.153 ± 1.311	4.210 ± 1.632	4.100 ± 2.088	3.977 ± 0.553

Mean SD values were calculated for each group

*ALT* alanine aminotransferase, *AST* aspartate aminotransferase, *ALP* alkaline phosphatase, *TP* total protein, *ALB* albumin, *GLB* globulin, *TBIL* total bilirubin, *DBIL* bilirubin direct, *IBIL* indirect bilirubin

^*^ The statistical difference between each group and the normal group. ^#^ The statistical difference between each group and the control group. *, ^#^ P<0.05

**Table 3:** Renal function results test in blood at 3 months postoperatively

Detection index	Unit	Normal group	Control group	Material group	MSCs group
BUN	mmol/L	7.647 ± 1.540	9.233 ± 3.682	7.247 ± 2.116	11.227 ± 4.190
CR	mmol/L	69.707 ± 7.026	103.413 ± 27.201	94.840 ± 15.769	107.587 ± 27.686
UA	mmol/L	31.467 ± 2.155	30.733 ± 1.126	29.600 ± 0.141	29.700 ± 0.000

Mean SD values were calculated for each group

*BUN* blood urea nitrogen, *CR* creatinine, *UA* uric acid

**Table 4:** Blood routine test results at 6 months postoperatively

Detection index	Unit	Normal group	Control group	Material group	MSCs group
RBC	10^12^/L	5.467 ± 0.364	4.927 ± 0.190	4.250 ± 0.964	5.433 ± 0.233
HCT	%	36.067 ± 1.636	36.400 ± 4.369	26.933 ± 5.949	33.867 ± 2.014
RDW-CV	10^9^/L	13.200 ± 0.356	12.567 ± 0.865	12.567 ± 1.096	12.833 ± 0.759
RDW-SD	%	30.967 ± 2.253	31.433 ± 2.782	28.600 ± 2.128	29.300 ± 1.203
MCV	fL	66.133 ± 3.642	70.400 ± 2.264	63.500 ± 0.920	64.000 ± 2.551
HBG	g/L	123.000 ± 2.944	122.000 ± 10.801	92.667 ± 18.571	115.333 ± 6.944
MCH	pg	22.600 ± 0.990	23.667 ± 0.732	21.967 ± 0.713	21.800 ± 0.920
MCHC	g/L	342.000 ± 8.832	336.000 ± 10.614	345.667 ± 7.587	340.667 ± 1.886
WBC	10^9^/L	10.727 ± 0.281	8.347 ± 0.241	9.167 ± 2.172	10.777 ± 1.829
LYM#	10^9^/L	3.700 ± 0.009	2.299 ± 0.544	2.908 ± 0.603	3.947 ± 0.737^#^
LYM%	%	34.517 ± 0.926	27.457 ± 5.857	32.310 ± 4.066	36.503 ± 0.922
NEUT#	10^9^/L	6.165 ± 0.287	5.265 ± 0.285	5.518 ± 1.541	5.913 ± 0.946
NEUT%	%	57.443 ± 1.217	63.157 ± 4.320	59.440 ± 4.470	55.020 ± 1.007
MONO#	10^9^/L	0.566 ± 0.024	0.580 ± 0.134	0.565 ± 0.106	0.557 ± 0.101
MONO%	%	5.280 ± 0.204	6.977 ± 1.724	6.390 ± 1.289	5.167 ± 0.118
EO#	10^9^/L	0.264 ± 0.088	0.169 ± 0.012	0.158 ± 0.110	0.330 ± 0.045
EO%	%	2.470 ± 0.853	2.000 ± 0.163	1.643 ± 0.845	3.090 ± 0.127
BASO	10^9^/L	0.032 ± 0.013	0.017 ± 0.004	0.019 ± 0.002	0.023 ± 0.009
BASO%	%	0.360 ± 0.060	0.175 ± 0.025	0.217 ± 0.029	0.220 ± 0.057
PLT	10^9^/L	137.333 ± 13.695	148.667 ± 20.997	156.333 ± 39.878	126.333 ± 8.380
PDW	%	15.800 ± 0.163	15.833 ± 0.403	15.733 ± 0.205	16.233 ± 0.125
MPV	fL	7.167 ± 0.189	6.867 ± 0.205	6.800 ± 0.294	6.900 ± 0.141
PLCR	%	13.400 ± 1.349	11.367 ± 1.247	11.467 ± 3.055	13.500 ± 1.980
PCT	%	0.027 ± 0.012	0.030 ± 0.014	0.040 ± 0.029	0.017 ± 0.005
CRP	mg/l	4.373 ± 1.426	3.990 ± 0.670	14.663 ± 12.212	3.957 ± 0.651

Mean SD values were calculated for each group

*RBC* red blood cell, *HCT* hematocrit, *RDW-CV* red blood cell volume distribution width, *RDW-SD* red blood cell distribution width, *MCV* mean corpuscular volume, *HBG* haemoglobin, *MCH* mean corpuscular haemoglobin, *MCHC* mean corpuscular haemoglobin concentration, *WBC* white blood cell, *LYM* lymphocyte, *NEUT* neutrophile granulocyte, *MONO* monocyte, *EO* eosinophil, *BASO* basophil, *PLT* platelet, *PDW* platelet distribution width, *MPV* mean platelet volume, *PLCR* platelet-large cell ratio, *PCT* platelet volume, *CRP* C reactive protein

**Table 5:** Liver function test results in blood at 6 months postoperatively

Detection index	Unit	Normal group	Control group	Material group	MSCs group
ALT	IU/L	72.733 ± 17.093	122.400 ± 102.499	50.700 ± 58.432	50.800 ± 10.968
AST	IU/L	23.567 ± 1.126	32.167 ± 14.290	15.400 ± 11.052	23.400 ± 0.294
ALP	IU/L	60.567 ± 10.076	59.933 ± 25.986	39.800 ± 20.467	58.567 ± 4.615
TP	g/l	62.000 ± 0.648	62.433 ± 3.058	58.633 ± 21.150	60.167 ± 2.829
ALB	g/l	40.600 ± 1.283	38.400 ± 5.233	35.367 ± 12.761	38.800 ± 2.765
GLB	g/l	21.367 ± 1.698	24.033 ± 2.323	23.300 ± 7.763	21.333 ± 3.583
A/G		1.917 ± 0.222	1.633 ± 0.349	1.540 ± 0.554	1.893 ± 0.438
TBIL	Umol/L	4.807 ± 0.813	6.710 ± 1.714	6.720 ± 0.070	3.570 ± 0.536
DBIL	Umol/L	2.123 ± 0.229	4.057 ± 1.500	3.557 ± 1.415	2.333 ± 0.352
IBIL	Umol/L	2.683 ± 0.877	2.653 ± 0.476	2.160 ± 0.854	1.237 ± 0.189

Mean SD values were calculated for each group

*ALT* alanine aminotransferase, *AST* aspartate aminotransferase, *ALP* alkaline phosphatase, *TP* total protein, *ALB* albumin, *GLB* globulin, *TBIL* total bilirubin, *DBIL* bilirubin direct, *IBIL* indirect bilirubin

**Table 6:** Renal function results test in blood at 6 months postoperatively

Detection index	Unit	Normal group	Control group	Material group	MSCs group
BUN	mmol/L	8.763 ± 0.403	8.570 ± 1.241	8.263 ± 2.780	7.987 ± 1.025
CR	mmol/L	99.877 ± 7.261	100.123 ± 4.054	88.000 ± 32.339	89.457 ± 8.307
UA	mmol/L	33.633 ± 3.175	30.067 ± 1.819	30.000 ± 10.322	29.133 ± 0.694

Mean SD values were calculated for each group

*BUN* blood urea nitrogen, *CR* creatinine, *UA* uric acid

Figure 1 shows the expression levels of BGP at 3 or 6 months after surgery. At 3 months, the BGP results were lower in the control group (27.650 ± 2.405 μg/L) compared to the normal group (31.067 ± 3.279 μg/L), whilst the material (30.333 ± 4.794 μg/L) and MSCs groups (30.027 ± 3.387 μg/L) were not significantly different from the normal group (Fig. 1a). BGP content imbalance due to bone material implantation was not observed in the material and MSCs groups at 3 months after surgery. At 6 months, the BGP content in the material group (24.627 ± 3.262 μg/L) decreased, whilst that in the MSCs group (32.667 ± 1.597 μg/L) was higher compared to the normal group (Fig. 1b). In the normal group (30.087 ± 2.036 μg/L), the BGP content was stable at approximately 30 µg/L in both periods. The BGP content in the control group (29.99 ± 1.677 μg/L) increased at 6 months and was close to that in the normal group. We speculate that bone absorbency increased after bone removal, which tended to be stable over time. The BGP content in the material group was close to that in the normal group at 3 months and lower at 6 months. Due to the high bone conversion of collagen scaffold materials, when bone absorption is greater than bone formation, this leads to a decrease in the BGP content. The BGP content of the MSCs group was slightly higher than that of the normal group due to the continuous effect of bone formation. We hypothesised that HUC-MSCs could prolong bone repair time. This indicated that neither bone collagen granule material nor HUC-MSCs were toxic, and that HUC-MSCs could reduce inflammatory reactivity.

### Imaging analysis

#### Results of general observation

**Fig. 2:**
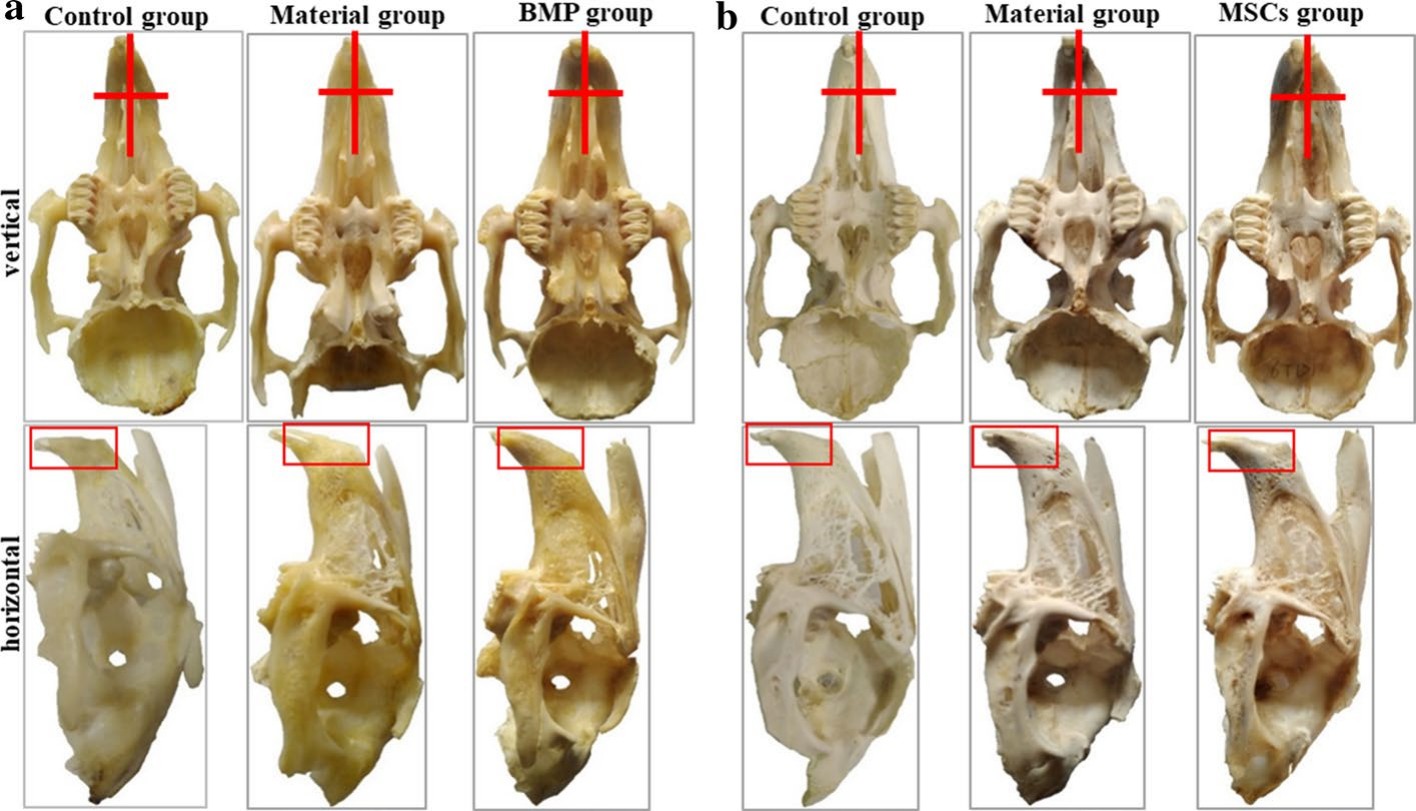
The general appearance of the skull and the red box is the surgical area. **a** Sampling group 3 months after surgery; **b** sampling group 6 months after surgery. The red box and red plus sign assist in displaying the recovery of the transplanted area

The appearance of the skull was observed from both vertical and horizontal angles, with the surgical position in the red box (Fig. 2). From a vertical point of view, asymmetry of the left and right maxillary bones was observed in both the control group and the material group, whilst no significant asymmetry was observed in the MSCs group. From a horizontal point of view, the area of new bone in both the material and the MSCs groups was higher at 6 months than at 3 months. In the control group, there were no obvious changes in the surgical location during the two periods.

#### Micro‑CT imaging

**Fig. 3:**
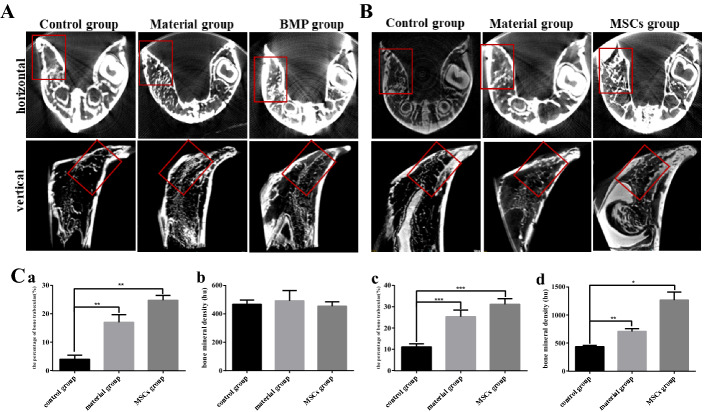
Micro-CT results. **A** CT images from different angles at 3 months. **B** CT images from different angles at 6 months; **C-a** the percentage of bone trabeculae at 3 months after surgery; **C-b** bone mineral density at 3 months after surgery; **C-c** the percentage of bone trabeculae at 6 months after surgery; **C-d** bone mineral density at 6 months after surgery. All groups were compared with the control group, and the difference was expressed as asterisk. *p < 0.05, **p < 0.01 or ***p < 0.001. The red box represents the surgical area

The internal images of the normal side and the operative side of each group were compared at 3 or 6 months for preliminary analysis (Fig. 3A, B). The red box represents the surgical site, with accurate analysis of bone density and the percentage of trabecular bone in each group (Fig. 3C). Internal images of both angles showed no significant repair of the surgical side in the control group during the two periods. A small amount of new bone tissue was found on the surgical side of the material group. A significant amount of new bone tissue was found on the surgical side of the MSCs group 6 months after surgery. The results showed that the percentage of bone trabeculae was highest in the MSCs group (24.79 ± 1.22%), followed by the material group (16.98 ± 2.21%) and the lowest in the control group (3.95 ± 1.03%) at 3 months after surgery (Fig. 3Ca). The results at 6 months (Fig. 3Cc) were similar to those at 3 months (MSCs group: 31.18 ± 2.12%, material group: 25.29 ± 2.53%, control group: 11.05 ± 1.23%). The bone mineral density (BMD) of the control (467.63 ± 24.27 hu), material (492.4 ± 58.74 hu) and MSCs groups (454.17 ± 25.18 hu) was not significantly different at 3 months (Fig. 3Cb). The BMD of MSCs group (1268.8 ± 116.73 hu) was significantly higher than that of the other two groups (material group: 706.67 ± 39.84 hu, control group: 435.27 ± 17.92 hu) at 6 months (Fig. 3Cd). Therefore, the osteogenic ability of bone collagen particles combined with HUC-MSCs was significantly better than that of bone collagen particles alone.

### Histological analysis

#### Hematoxylin–eosin staining

**Fig. 4:**
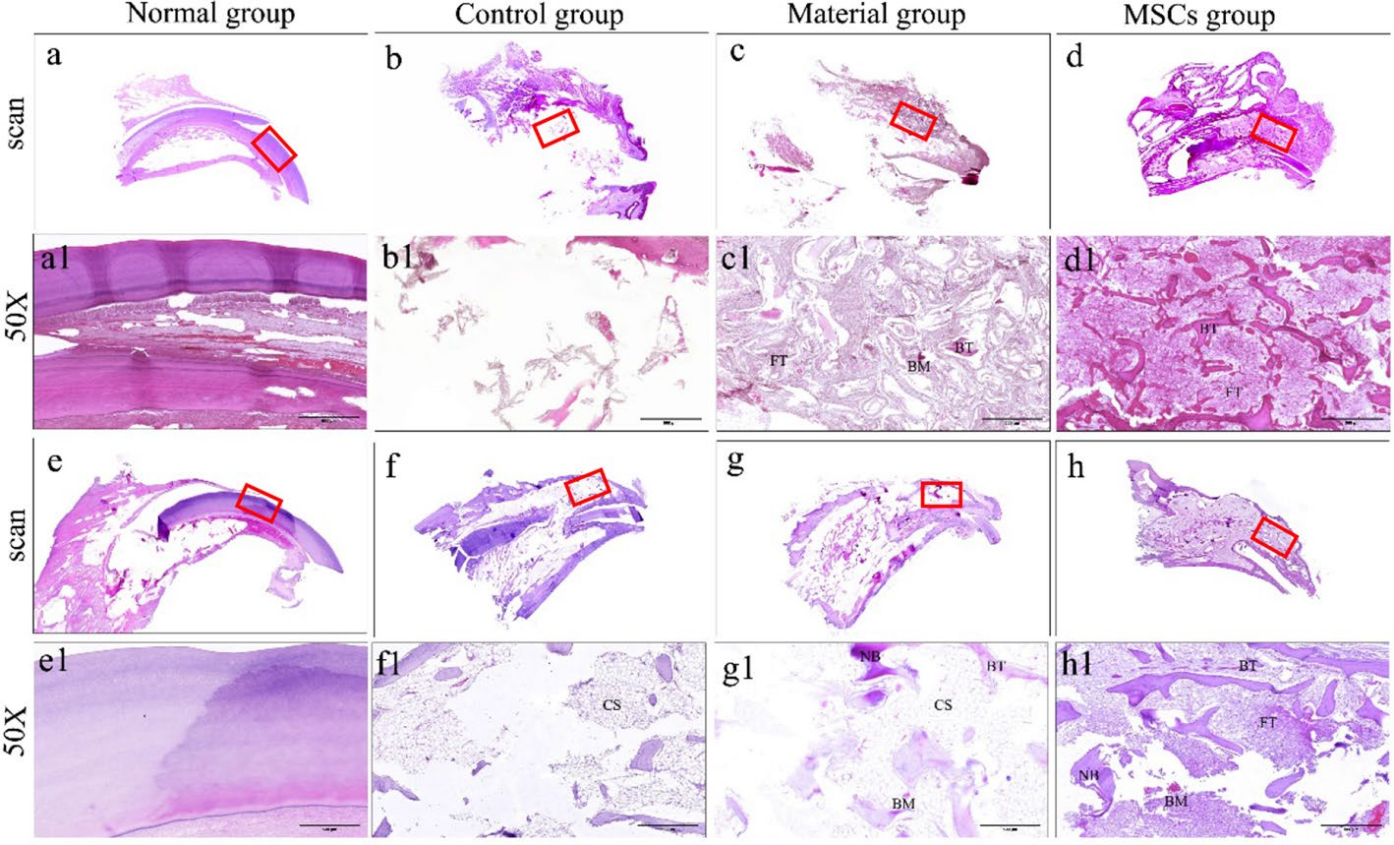
HE staining results. **a**–**d**, **a1**–**d1** Sampling group 3 months after surgery; **e**–**h, e1**–**h1** sampling group 6 months after surgery. **a**, **e**, **a1**, **e1** Normal group. **b**, **f**, **b1**, **f1** Control group. **c**, **g**, **c1**, **g1** Material group. **d**, **h**, **d1**, **h1** MSCs group. **a**–**h** The scan results of HE staining. **a1**–**h1** The result of HE staining after 50 times magnification. *BM* bone marrow, *FT* fibrous tissue, *BT* bone trabecula, *NB* new bone, *CS* cavitation structure

Hematoxylin–eosin (HE) staining showed that the normal incisor bone was a uniform bone matrix (Fig. 4a1, e1). The bone defect area without any implanted material showed no new bone formation at neither periods, with only a few scattered bits of bone (Fig. 4b1, f1). Three months after the implantation of bone collagen particle alone, a large number of bone fibres and a small amount of bone marrow and trabeculae were observed in the bone defect area (Fig. 4c1). After 6 months, only a small amount of bone marrow and trabeculae were observed in the bone defect area, with the rest appearing as cavitation structures (Fig. 4g1). Three months after the implantation of bone collagen particles in combination with HUC-MSCs, a large number of trabeculae and fibrous tissues were observed in the bone defect area (Fig. 4d1). After 6 months, a large new bone formation was visible in the bone defect area (Fig. 4h1).

#### Sirius red staining

**Fig. 5:**
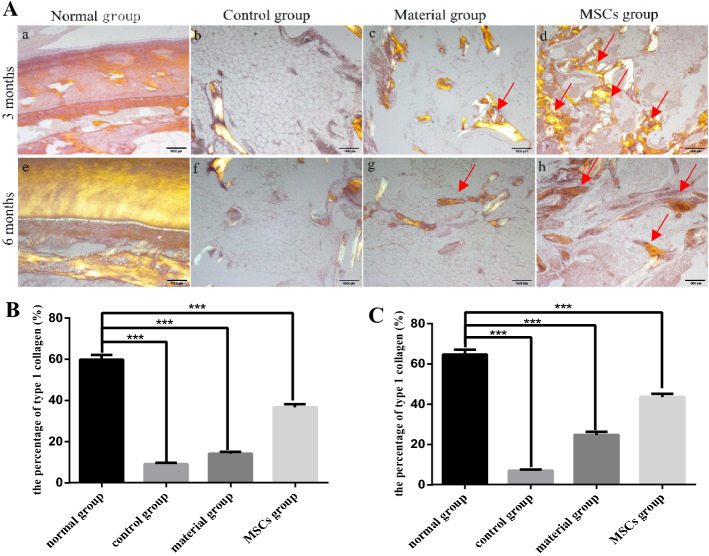
Sirius red staining results (×40). **Aa**–**d** Sampling group 3 months after surgery; **e**–**h** sampling group 6 months after surgery. **a**, **e** Normal group. **b**, **f** Control group. **c**, **g** Material group. **d**, **h** MSCs group. Mark the area of the positive signal with a red arrow. **B** The percentage of type 1 collagen in each group at 3 months after surgery. **C** The percentage of type 1 collagen in each group at 6 months after surgery. All groups were compared with the Normal group, and the statistical difference was denoted by asterisk. ***p < 0.001

**Fig. 6:**
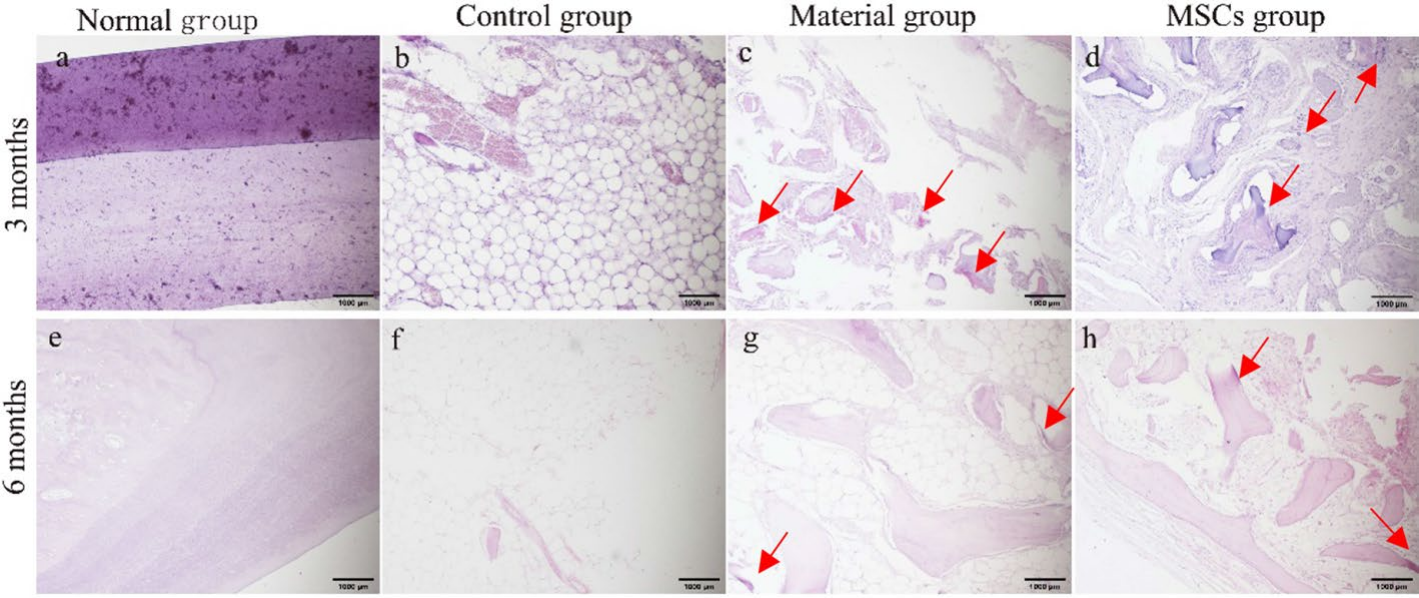
PAS staining results (×100). **a**–**d** Sampling group 3 months after surgery; **e**–**h** sampling group 6 months after surgery. **a**, **e** Normal group. **b**, **f** Control group. **c**, **g** Material group. **d**, **h** MSCs group. Mark the area of the positive signal with a red arrow

Type 1 collagen is stained bright orange by Sirius red staining. Sirius red staining showed high levels of collagen type 1 were high and evenly distributed in the normal incisor bone (Fig. 5Aa, e), compared to the control group, in which only a minimal amount of type 1 collagen was present in the scattered bones (Fig. 5Ab, f). Only a small amount of collagen type 1 was observed in the bone defect area after the implantation of bone collagen particles alone (Fig. 5Ac, g). However, after the implantation of bone collagen particles combined with HUC-MSCs, a large amount of collagen type 1 was visible in the bone defect area (Fig. 5Ad, h). Compared with the normal group, the content of collagen type 1 in each group was statistically different at 3 months (Fig. 5B) and 6 months (Fig. 5C) after surgery.

#### Periodic acid–Schiff staining

Periodic acid–Schiff (PAS) is used to stain chondrocytes a dark purple or crimson colour. The results of PAS staining showed that the normal incisor bone was a uniform bone matrix without chondrocytes (Fig. 6a, e). In the control group, a large number of cavitation structures were observed without marked dark purple or crimson areas (Fig. 6b, f). A small amount of dark purple or crimson areas was visible in the bone defect area after the implantation of bone collagen particles alone, indicating the presence of a small amount of chondrocytes (Fig. 6c, g). After the implantation of bone collagen particles combined with HUC-MSCs, a large number of dark purple or crimson areas appeared in the bone defect area, indicating the presence of a large number of chondrocytes (Fig. 6d, h).

#### Alkaline phosphatase staining

**Fig. 7:**
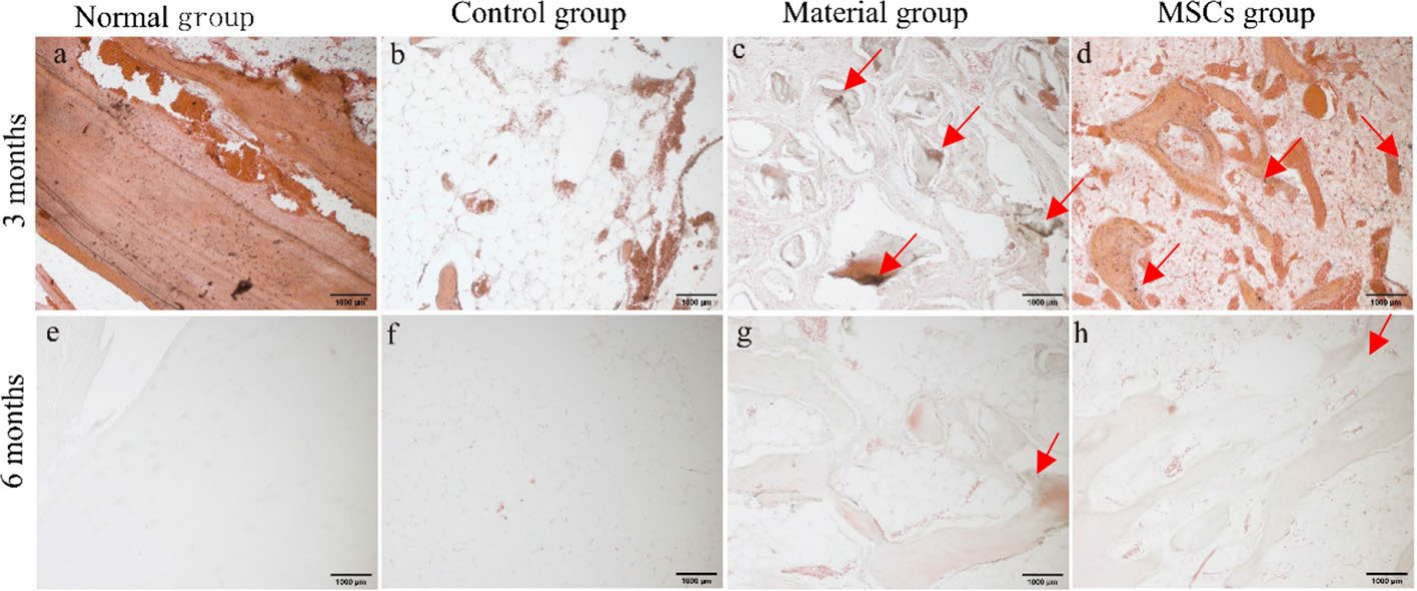
ALP staining results (×100). **a**–**d** Sampling group 3 months after surgery; **e**–**h** sampling group 6 months after surgery. **a**, **e** Normal group. **b**, **f** Control group. **c**, **g** Material group. **d**, **h** MSCs group. Mark the area of the positive signal with a red arrow

Alkaline phosphatase (ALP) staining can indirectly stain osteoblasts black. ALP staining showed that the normal incisor bone was a uniform bone matrix with no osteoblast structure (Fig. 7a, e). The bone defect in the control group was a cavitation structure, and osteoblast structure was not observed (Fig. 7b, f). Several black spots were observed in the bone defect area of the material and MSCs groups at 3 months (Fig. 7c, d), and the number of black spots decreased at 6 months (Fig. 7g, h), indicating that the number of osteoblasts varied from high to low.

#### BMP-2 immunohistochemistry (IHC) results

**Fig. 8:**
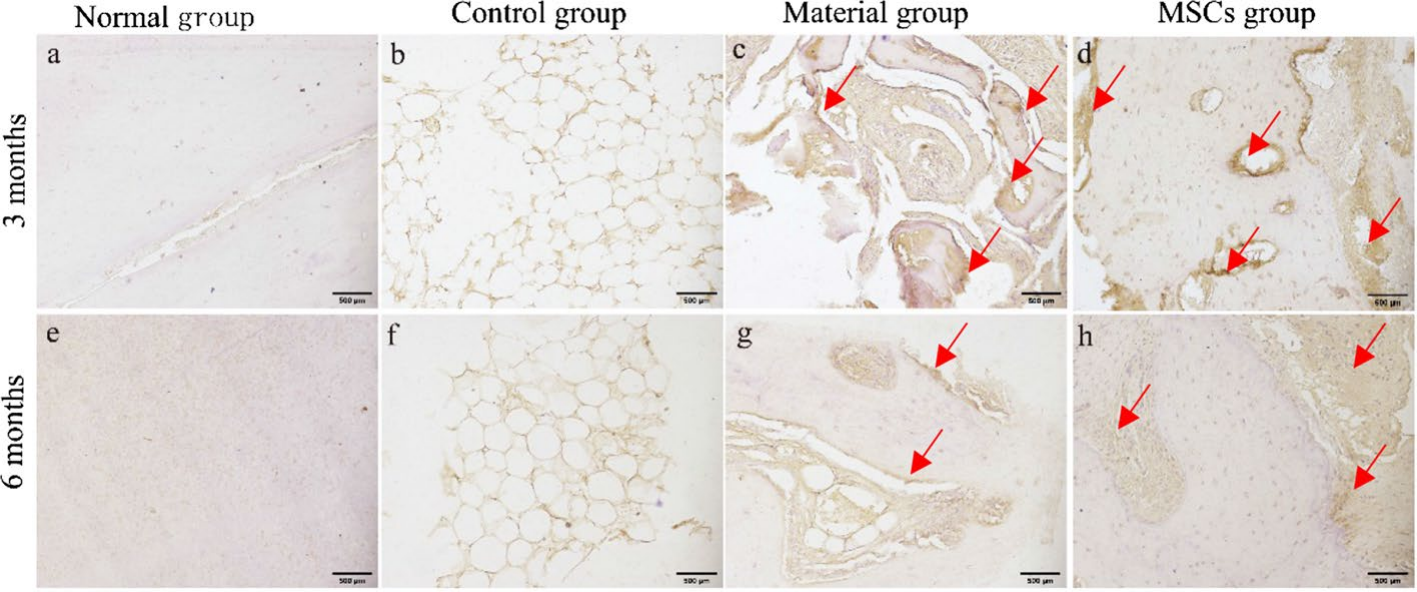
IHC results of BMP-2 (×200). **a**–**d** Sampling group 3 months after surgery; **e**–**h** sampling group 6 months after surgery. **a**, **e** Normal group. **b**, **f** Control group. **c**, **g** Material group. **d**, **h** MSCs group. Mark the area of the positive signal with a red arrow

It has been reported that BMP-2 plays a pivotal role in bone formation. Overexpression of BMP-2 is involved in regulating the formation and remodelling of mineralised tissue [26]. As shown in Fig. 4, BMP-2 expression was not detected in the normal group and the control group. However, BMP-2 positive cells were detected in the sections of the material group and the MSCs group. In the material and the MSCs groups, BMP-2 was mainly expressed in osteocytes and osteoclasts at the edge of trabecular bone (Fig. 8c, d, g, h). The expression level of BMP-2 in the material and the MSCs groups at 3 months after surgery was significantly higher than that at 6 months after surgery. The expression level of BMP-2 was the highest in the MSCs group. The results showed that the ability of active collagen particles combined with HUC-MSCs to induce the generation of BMP-2 was better than that of bone collagen particles alone.

### Proliferation and apoptosis analyses

**Fig. 9:**
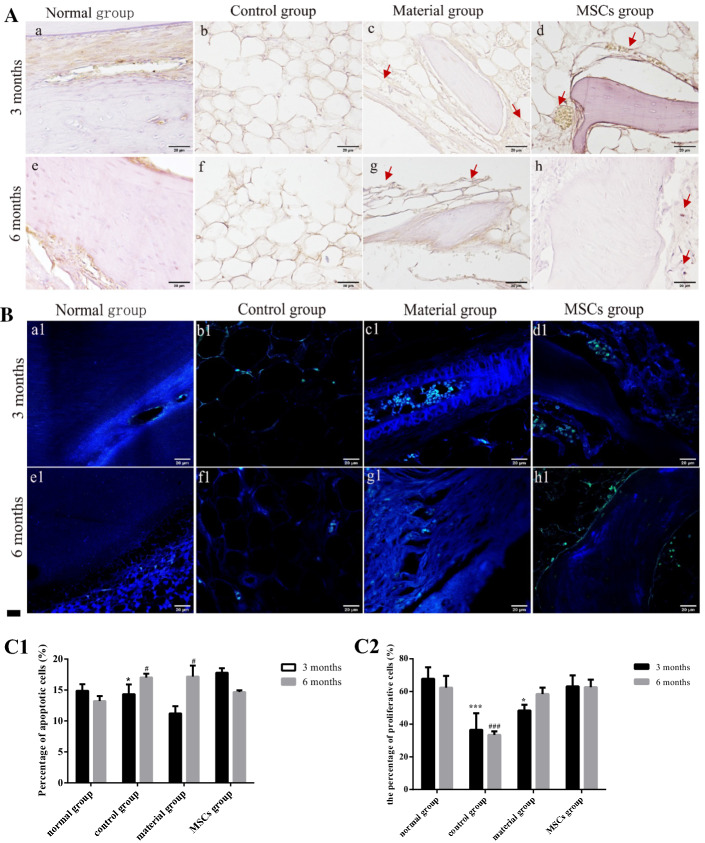
**A** TUNEL results (×400). **B** Ki67 results (×400). **a**–**d**, **a1**–**d1** Sampling group 3 months after surgery. **e**–**h**, **e1**–**h1** Sampling group 6 months after surgery. **a**, **e**, **a1**, **e1** Normal group. **b**, **f**, **b1**, **f1** Control group. **c**, **g**, **c1**, **g1** Material group. **d**, **h**, **d1**, **h1** MSCs group. **C1** Percentage of apoptotic cells in each group at 3 or 6 months. **C2** Percentage of proliferative cells in each group at 3 or 6 months. Cells labelled in green represent cells that are proliferating. Asterisk represents the statistical difference between each group and the normal group at 3 months. Hash represents the statistical difference between each group and the normal group at 6 months. *, ^#^*p *< 0.05 or ***, ^###^*p* < 0.001

TdT-mediated dUTP nick-end Labelling (TUNEL) (Fig. 9A) methods and antihuman Ki67 (Fig. 9B) staining were used to detect cell proliferation and apoptosis in each group at 3 and 6 months after surgery. TUNEL assay (Fig. 9C1) showed that the percentage of apoptosis cells of the control group (14.32 ± 1.29%) was similar to that of the normal group (14.87 ± 0.75%) of 3 months after surgery. The percentage of apoptosis cells of the material group (11.21 ± 0.84%) was lower than that of the normal group. The percentage of apoptosis cells of the MSCs group (17.79 ± 0.52%) was significantly higher than that of the normal group. At 6 months after surgery, the percentage of apoptosis cells of the control group (17.03 ± 0.51%) and the material group (17.18 ± 1.24%) increased and was higher than that of the normal group (13.23 ± 0.57%). The percentage of apoptosis cells of the MSCs group (14.68 ± 0.19%) decreased and was close to that of the normal group. The immunohistochemical test results of Ki67 (Fig. 9C2) showed that the percentage of proliferative cells of the control (36.56 ± 8.26%) and the material groups (48.39 ± 2.89%) was significantly lower than that of the normal group (67.84 ± 5.73%) at 3 months after surgery. The percentage of proliferative cells of the MSCs group (63.17 ± 5.5%) was similar to that of the normal group. At 6 months after the surgery, there was no significant change in the percentage of proliferative cells of the control group (33.49 ± 1.75%), which was still lower than that of the normal group (62.34 ± 5.89%). The percentage of proliferative cells of the material group (58.52 ± 3.2%) increased markedly but did not exceed that of the normal group. The percentage of proliferative cells of the MSCs group (62.68 ± 3.78%) remained close to that of the normal group.

## Discussion

Compared with autologous MSCs, allogeneic MSCs have different degrees of ethical problems. The ethical issue of human embryonic-derived mesenchymal stem cells is the most controversial. HUC-MSCs, which are obtained from neonatal umbilical cords, have various advantages, including their abundance, low cost, and safety. Neonatal umbilical cords are considered medical waste, such that the use of HUC-MSCs is more ethical than stem cells obtained from embryo [22]. This makes them an ideal candidate for medical applications [27]. Although HUC-MSCs have good bone induction, they are easily absorbed and degraded in vivo. Collagen is one of the most widely used bone-filling biomaterials in bone tissue engineering [28]. However, studies have shown that the function of collagen-based biomaterials in bone repair alone is limited [29]. Collagen scaffold material is an ideal scaffold material to enhance the action of HUC-MSCs on bone defect sites [24]. The combination of collagen scaffolds can slow down the degradation rate of HUC-MSCs, thus prolonging the bone repair time.

The bone collagen particles used in this study were obtained from heterogeneous bone namely bovine cancellous bone, after degreasing and decellularisation. Bone matrix particles are mainly composed of hydroxyapatite and collagen. This material has high strength and strong bone conductivity. The bone matrix retains a natural three-dimensional network that facilitates cell implantation and growth. After decellularisation, the allogeneic bone can effectively reduce its immunogenicity [30]. The heterogeneous bone matrix is more widely derived than allograft bone, and has a shorter degradation and absorption time, thus meeting the requirements of an ideal carrier. Newly formed bone tissue was identified in the defect areas of MSCs group by micro-CT and various staining methods. As expected, the MSCs group was more effective than the material group. To the best of our knowledge, this study is the first to evaluate the effects of bone collagen particles combined with HUC-MSCs on incisor bone regeneration at 3 and 6 months after surgery.

Although HUC-MSCs have been successfully used in the treatment of various bone lesions in vivo [31–33], the environment of the incisor is quite different from other sites in terms of the force, stress and movement of the alveolar bone. Therefore, the therapeutic strategy for alveolar bone defects needs to be re-evaluated.

The purpose of this study was to evaluate the effect of bone collagen particles combined with HUC-MSCs on the repair of alveolar bone defects. Serological detection allows for the comprehensive characterisation of body function, which is essential in the assessment of the biocompatibility of bone collagen particles and HUC-MSCs in vivo. Routine blood tests are often used to detect inflammation and early disease. Liver function tests can be used to detect liver injury. Kidney function testing is used to assess kidney function, which can in turn be used to evaluate the state of the kidney health. C-reactive protein is highly sensitive to infectious inflammation [34]. BGP is directly produced and released from osteoblasts, and is positively correlated with bone formation. If the biocompatibility of the material is good, the blood indicators will not be significantly different from that of the normal group. Here, the results of the blood test showed that the bone collagen particles and HUC-MSCs had good biocompatibility, and that the HUC-MSCs could reduce the inflammatory response. We speculated that bone collagen particles and HUC-MSCs do not significantly change the physiological environment of the body during absorption, similar to metal alloy materials [35–37]. In addition, the main components of bone collagen particles are hydroxyapatite and collagen type 1, which can effectively reduce its immunogenicity. Studies have found that HUC-MSCs isolated from Wharton’s jelly did not express HLA-QPDQDR, nor co-stimulators CD80 and CD86 [23]. This indicated HUC-MSCs have low potential to activate immune cell response. The gross observation of the skull model and the micro-CT scan results showed that the effect of bone collagen particles combined with HUC-MSCs on bone regeneration and repair was stronger than that of bone collagen particles alone. Tissue staining results also showed that the combination of bone collagen particles with HUC-MSCs had significantly increased the trabecular bone formation rate. Studies have shown that the formation of new bone depends on bone trabecular density and connection rate [38]. The number of osteoblasts and chondrocytes was also found to increase significantly. The expression levels of collagen 1 were significantly higher than those in the material group. The results of cell proliferation and apoptosis analyses suggested that the combination of bone collagen particles with HUC-MSCs promotes cell proliferation and apoptosis, thus promoting bone regeneration.

Bone induction refers to the induction of connective tissue adjacent to bone graft by certain bone growth factors of bone materials. By acting on undifferentiated bone progenitor cells and promoting their differentiation and proliferation, these cells eventually become osteoblasts and promote the formation of new bone [39]. The alveolar cleft model established in this study comprised the formation of a gap by pulling out the incisors [9]. After the remove of the incisors, no evident damage to the inner wall of the bone around the incisors was observed, except for the root of the incisors. Therefore, the osteogenic induction ability of different positions was not uniform after the addition of bone collagen particles. Although a gap remained between the newly generated bone, from the action of bone collagen particles combined with HUC-MSCs and the normal incisor, it was enough to prove that HUC-MSCs could be used for bone generation inducers combined with bone materials for bone regeneration and repair. In the future, this model may be used for tissue engineering bone regeneration, with potential for use in clinical applications.

## Conclusions

With the development of tissue engineering technology, it is difficult to achieve bone repair using scaffold materials alone. The combination of HUC-MSCs with biomaterials represents a promising strategy in the field of regenerative medicine and bone repair. In this study, the effect of bone collagen particles combined with HUC-MSCs on bone repair and regeneration was found to be markedly more effective than that of bone collagen particles alone. As such, using a combination of HUC-MSCs and bone collagen particles to fill a bone defect site represents a simple and rapid method suitable for the treatment of alveolar cleft bone defects, as well as a promising method for the reconstruction of incisor bone defects.

## Materials and methods

### Isolation and culture of HUC-MSCs

HUC-MSCs were isolated from Wharton’s jelly, using tissue block adherent culture. The blood vessels in the human umbilical cord were removed and the tissue cut into 1 mm^3^ pieces. The tissue blocks were inoculated in 10-cm petri dishes and incubated upside down for 4 h at 37 °C in 5% CO_2_. After fixing the tissue blocks to the bottom of the plates, α-MEM complete medium was added to the primary culture. After approximately 2 weeks, the cells had spread in a radial manner. The cell suspensions were collected and centrifuged in a 15-mL centrifuge tube at 800 r/min for 5 min. The supernatant was discarded and gently blown into a single-cell suspension for subculture. After five generations, HUC-MSCs were collected.

### Preparation of implant materials

Bone collagen particles were obtained by the decellularisation and degreasing of bovine cancellous bone (Haiao, Yantai Zhenghai Bio-Tech Co., Ltd, Shandong, China). The main components of bone collagen particles are hydroxyapatite and collagen 1, which effectively reduce their immunogenicity. The particles also preserve the natural structure of bone, allowing for the growth of cells and blood vessels. The bone collagen particles were provided by Zhenghai Biotechnology Co. (Yantai, China). Cells within 5 generations were selected for inoculation with bone collagen particles and cultured in a carbon dioxide incubator for 0.5 h. The concentration of HUC-MSCs should reach 10^7^ cells/mL.

### Surgical procedure and treatment

One- to two-month-old JWRs were purchased from Huafukang Biotechnology Co. (Beijing, China). A total of 24 female JWRs (bodyweight: 2000 ± 300 g) were used in this study. All rabbits were kept in the animal room of the National Research Institute for Family Planning, with free access to water and food, at a temperature of 23–25 °C, a humidity of 50–60%, noise levels under 60 dB, and a 12-h light/dark cycle. The animal room was clean, dry, and ventilated. The study was approved by the local research and ethics committee.

**Fig. 10:**
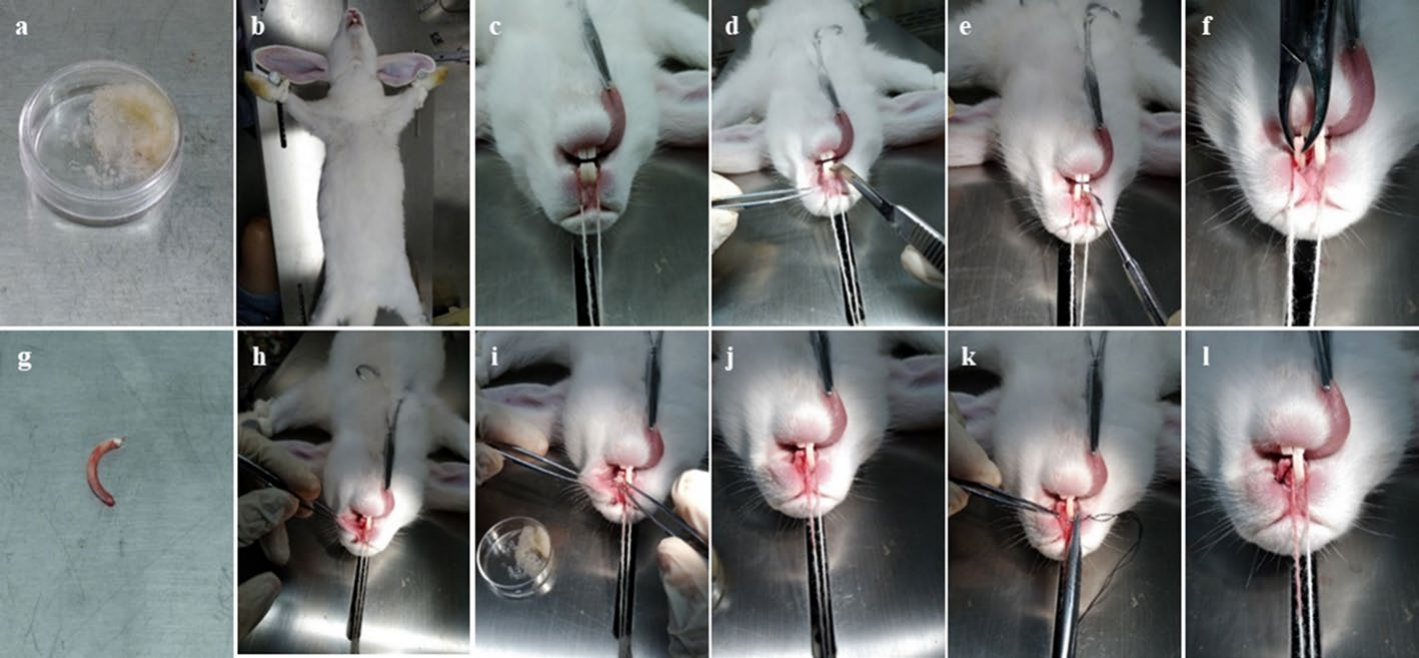
Surgical process: **a** collagen granules were incubated by HUC-MSCs. **b** Fix the anaesthetised rabbits. **c** Open the oral cavity after anaesthesia. **d**–**e** Incise the skin. **f**–**h** Remove the left incisor. **i**–**j** Add collagen particles. **k**–**l** Suture the skin

The rabbits were randomly divided into four groups (*n* = 6): normal, control, material and MSCs groups. They were anesthetised by intravenous injections of serazine hydrochloride (1–2 mg/kg). The model of alveolar clefts was established by removing the incisors on the left side of the upper jaw (Fig. 10f–h). The normal group was fed normally without surgery. In the control group, after removing the incisors, the collagen membrane was directly covered and the skin was sutured. In the material group, the gaps left after the removal of the incisors were filled with bone collagen particles, the collagen membrane was covered and the skin was sutured. In the MSCs group, the gaps were filled with bone collagen particles incubated with HUC-MSCs, the collagen membrane was covered and the skin was sutured. The rabbits were treated with antibiotics for 1 week after surgery to prevent infection. Blood was collected from each group at 3 and 6 months after surgery. Thereafter, the rabbits were euthanised and the upper jaw was examined and obtained for further evaluation.

### Blood analysis

Three months after the surgery, three rabbits were randomly selected from each group. Routine blood tests, as well as analyses for liver function, kidney function and BGP, were performed by collecting 3.5 mL of venous blood from the ears. After using 1 mL of whole blood for routine blood testing, the serum was isolated from the remaining 2.5 mL. These serum samples were used for blood biochemistry testing. Routine blood tests were performed using an LH 750 automated haematology analyser (Beckman Coulter, USA). The blood biochemistry test was performed using a DXC 800 automated biochemical analyser (Beckman Coulter, USA). At 6 months after surgery, the indexes of the three rabbits were detected.

### Micro-CT analysis

Three months after surgery, one rabbit was randomly selected from each group to create a skull model. The procedure was repeated 6 months after surgery. The skull was photographed to obtain a record of visualisation. Bone regeneration in the skull was evaluated using micro-computed tomography (CT). Bone regeneration in the alveolar cleft was evaluated using a micro-CT system (SIEMENS Inveon Research Workplace 4.2, Beijing). The three-dimensional repair of the injuries to each group was observed, and the trabecular bone and bone density values were recorded.

### Histology staining

Rabbits were euthanised and histologically evaluated at 3 or 6 months after surgery. The specimens were immersed in 4% paraformaldehyde for 24 h and decalcified in 10% EDTA. After decalcification, the tissue was embedded in paraffin for the preparation of 4-µm sections using a microtome.

The morphology of the cells was evaluated using hematoxylin and eosin (HE) staining. Hematoxylin stains the nuclei a blue-violet colour, whilst other tissues were stained red by eosin. The products of chondrocytes are metachromatic and can eventually differentiate into osteoblasts. Periodic Acid–Schiff stains (PAS) staining was used to dye chondrocytes a dark purple or crimson colour. Collagen type 1 is generally found in the bone and in tendon fibres, and can be dyed bright orange by Sirius red staining. Image J software was used to calculate the relative percentages of the positive staining areas in each section. Osteoblasts, which are markers of bone formation, were dyed black by alkaline phosphatase (ALP) staining for their visualisation for the location of osteoblasts.

Bone morphogenetic protein 2 (BMP2) is a marker of bone formation. Primary anti-BMP2 (ab6285, 1:1000 dilution; Abcam) and HRP-coupled secondary antibody were used to detect BMP2 in the tissue samples. Primary anti-Ki67 (ab15580, 1:1000 dilution; Abcam) was used to detect cell proliferation by immunofluorescence. For the detection of apoptotic cells using of TUNEL, three regions were randomly selected and the percentage of Ki67- and TUNEL-positive cells was quantified using Image J software.

### Statistical analysis

All values are expressed as the mean ± standard deviation (SD). *p *< 0.05 indicated statistical significance. Data were analysed statistically by factorial analysis of variance and Student’s *t* test in GraphPad Prism software (GraphPad Prism 6).

## Data Availability

All data generated or analysed during this study are included in this published article.

## Abbreviations

HUC-MSCs: Human umbilical cord mesenchymal stem cells
JWRs: Japanese white rabbits
micro-CT: Micro-focus computerised tomography
HE: Hematoxylin–eosin
ALP: Alkaline phosphatase
PAS: Periodic Acid–Schiff stain
IHC: Immunohistochemical
